# The Electronically Confined Space Analogy Elucidates How Second‐Row Triatomic 18‐Valence‐Electron Molecules Shape Life and Light

**DOI:** 10.1002/open.202500557

**Published:** 2026-03-10

**Authors:** Jordi Poater, Clara Viñas, Francesc Teixidor

**Affiliations:** ^1^ Departament de Química Inorgànica i Orgànica & IQTCUB Universitat de Barcelona Barcelona Spain; ^2^ ICREA Barcelona Spain; ^3^ Institut de Ciència de Materials de Barcelona Consejo Superior de Investigaciones Científicas Bellaterra Spain

**Keywords:** electronic structure theory, Electronically Confined Space Analogy, ozone photochemistry, planetary atmospheric chemistry, UV protection mechanisms

## Abstract

Our recently proposed Electronically Confined Space Analogy (ECSA) postulate offers a unified framework for interpreting the structure, stability, and photochemical behavior of 18‐valence‐electron (18‐VE) molecules composed of second‐row elements. We show that bent triatomic 18‐VE isomers, such as ozone, exhibit strong ultraviolet absorption due to delocalized π systems and low‐energy electronic transitions, whereas their cyclic counterparts are photochemically inert. Bent structures are consistently more stable than cyclic analogs, with stability governed by electronic polarization and symmetry rather than electronegativity. This thermodynamic preference ensures the dominance of UV‐absorbing species under atmospheric conditions. Applying ECSA provides a molecular‐level explanation for ozone's unique role as Earth's stratospheric UV filter, complementing and extending the traditional Chapman mechanism. We further propose a UV‐driven ozone cycle that incorporates excited states and intermediates, offering an improved description of ozone photophysics and atmospheric resilience.

## Graphical abstract

The Electronically Confined Space Analogy explains ozone's unique UV absorption by linking bent 18‐valence‐electron structures to enhanced stability and photochemical activity, offering a molecular‐level rationale for its dominance as Earth's stratospheric UV filter.

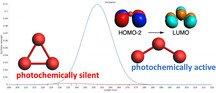

## 1 Introduction

Chemists are used to electron counting rules. These are the silent architects of chemical behavior guiding how atoms assemble into molecules and materials. These rules are rooted in quantum mechanics and empirical observations that define “magic numbers” of valence electrons (VEs) that confer stability or reactivity [1]. Well‐known rules are the Octet rule that is at the heart of main‐group chemistry [2], the 18‐Electron rule for low‐valent metals with π‐acceptor ligands [3], and the 16‐Electron rule that is common for square‐planar d^8^ complexes, the Hückel's rule (4n + 2 π‐electrons) [4], Wade's rule for boranes and metalloclusters [5], the Jemmis’ mno rule for condensed polyhedral clusters [6], the Zintl‐Klemm Concept that deals on electron transfer in intermetallics [7], the Mingos’ Isolated Pentagon rule for stabilized fullerenes [8], the Hirsch's Spherical Aromaticity that applies well for icosahedral clusters [9], the Quantum Foundations 2n^2^ rule [10], and the Polyhedral Skeletal Electron Pair Theory (PSEPT) [11], just to mention some of the most popular rules [12].

From our hand, some time ago, we proposed the Electronically Confined Space Analogy (ECSA) postulate [13, 14, 15], which states: *If a molecule or molecular fragment occupies a particular confined space with a specific number of valence electrons*, *then any other molecule or fragment with the same number of valence electrons can also exist in a comparable confined space.* ECSA was initially developed to demonstrate the equivalence between hydrogen derivatives of the neighboring elements boron and carbon—specifically, between boron hydrides and hydrocarbons—and to illustrate that arenes are analogous to *closo*‐boranes [13, 14, 15]. A carbon framework can be transformed—via virtual transmutation—into a boron hydride structure, often with one or more additional boron vertices. Now, we have primarily applied this concept to molecules and fragments composed of second‐row elements. Within this framework, isoelectronic molecules are considered ECSA isoelectronic, as they share both electronic and spatial characteristics.

**FIGURE 1:**
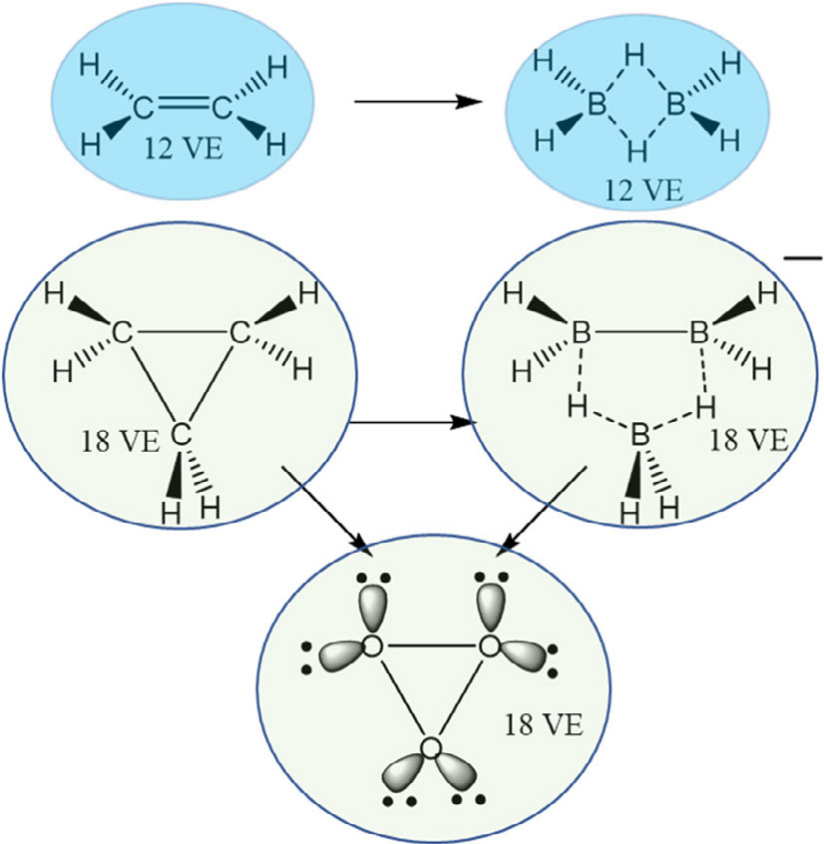
Illustration of the ECSA postulate using examples with 12 and 18 valence electrons, highlighting the resulting confined spatial arrangements.

Figure 1 exemplifies the Electronically Confined Space Analogy (ECSA) postulate by presenting isoelectronic molecules and ions that share both the same number of valence electrons and similar confined electronic spaces. For the 12‐valence electron (12‐VE) case, ethylene (C_2_H_4_) and diborane (B_2_H_6_) are shown; for the 18‐VE case, cyclopropane (C_3_H_6_), the octahydrotriborate anion ([B_3_H_8_]^−^), and cyclic ozone (O_3_) are depicted. In each example, circles highlight regions of confined space, illustrating how different molecular frameworks can accommodate the same number of electrons within analogous spatial constraints, as predicted by the ECSA postulate [13, 14, 15]. It is important to note that not all second‐row elements naturally possess enough valence electrons to achieve these configurations. In such instances, additional atoms—referred to as sacrificial atoms—are incorporated to supply the necessary electrons, thereby enabling the formation of ECSA isoelectronic molecules. These examples collectively demonstrate how the ECSA framework unifies structurally diverse molecules through their shared electronic and spatial characteristics. The ECSA postulate has been applied successfully to the generation of boron hydride structures from the structures of alkanes, and later it has allowed to prove that 2D and 3D aromatic structures respond to the same principles [15]. It has always been applied with success with the elements of the second period, the ones that matter for this work, while not much effort has been made for lower periods in the periodic table.

The aim of the present work is to analyze by means of ECSA a series of 18‐VE systems that are well‐characterized in coordination and organometallic chemistry [1], but their behavior in main‐group compounds featuring three second‐row atoms remains underexplored. In this work, we show that such 18‐VE systems can form paired structures in which one partner is photochemically inert (silent), whereas the other exhibits strong UV absorption due to a delocalized double bond. Without exception, the photochemically active species is the bent isomer, whereas the cyclic forms are photochemically inert.

## 2 Results and Discussion

### 2.1 The Curious Case of the Eighteen Valence Electrons with Three Second‐Row Atoms

**FIGURE 2:**
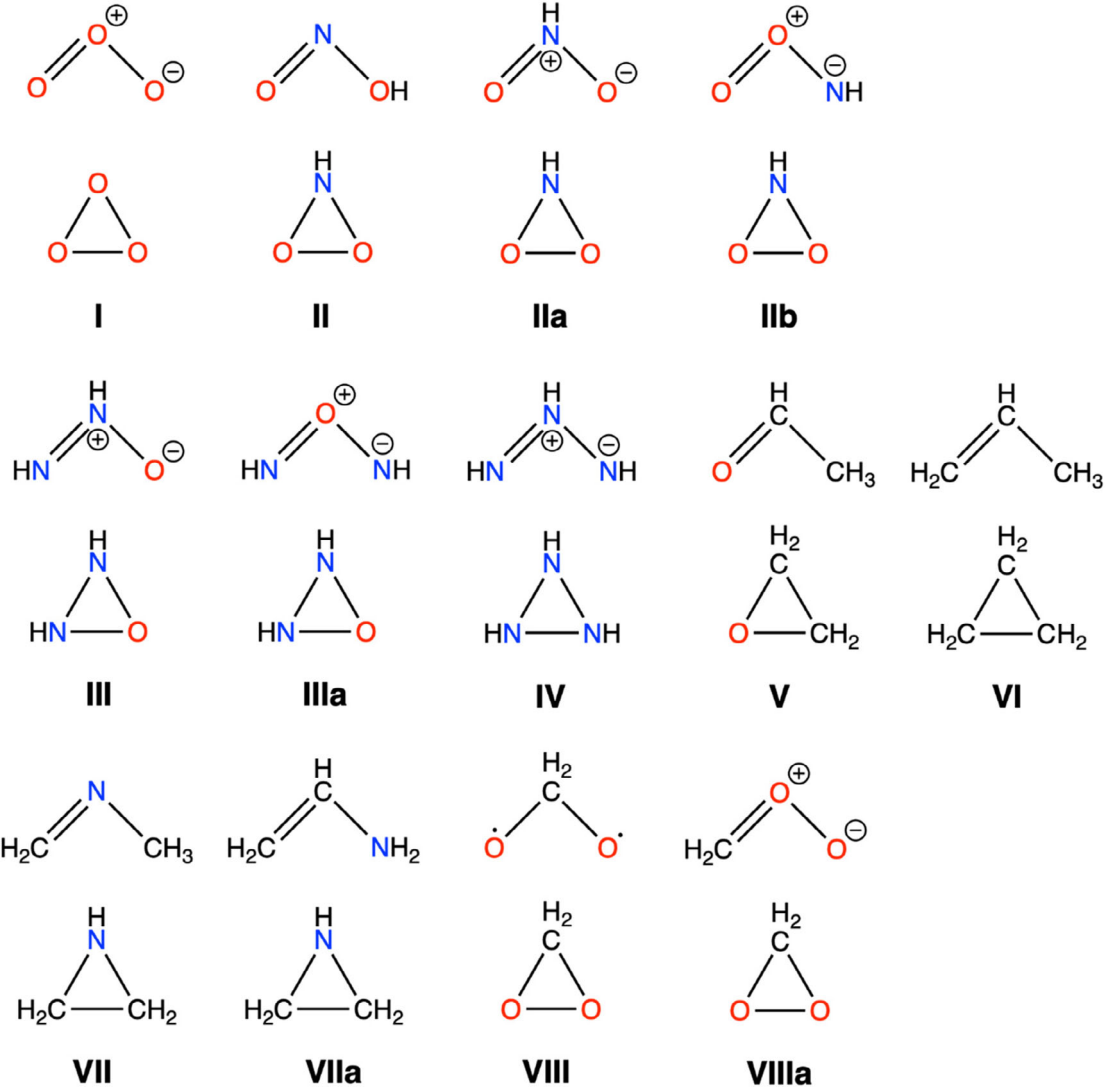
Series of alternate 18‐VE bent (top) and cyclic (bottom) systems under scrutiny.

First, we selected a series of molecules with three second‐row atoms that possess 18 VEs, as illustrated in Figure 2. Each molecule contains three second‐row elements and, when necessary, the 18‐VE count is achieved by either the addition of hydrogen atoms as sacrificial species or by introducing negative charges. In addition, we used protonated forms of the anionic molecules to ensure all of them are neutral. We must keep in mind that most of these diagrams in Figure 2 depict either individual resonance contributors or tautomeric forms, neither of which fully represent the true hybridized structure. The molecules shown in the upper part of the couples in Figure 2 are the ozone (O_3_, **I**), the nitrite anion (NO_2_^−^, **II**), the nitrous acid isomer (ON(H)O, **IIa**), the hypothetical aminodioxyl (O_2_NH, **IIb**), the hypothetical diimide‐N‐oxide (N_2_H_2_O, **III**), nitroxylamine (N_2_H_2_O, **IIIa**), triazene (H_3_N_3_, **IV**), acetaldehyde (H_4_C_2_O, **V**), the allyl anion (C_3_H_5_^−^, **VI**) in their protonated forms, the highly reactive N‐methylmethanimine (H_5_C_2_N, **VII**), ethenamine (H_5_C_2_N, **VIIa**), and the Criegee intermediate (OOCH_2_, **VIIIa**). Most of these adopt bent molecular geometries, although some of these structures depicted may not exist or could be intrinsically unstable. While the ECSA postulate focuses on valence electrons and comparable spatial confinement, it does not account for the specific chemical nature of the constituent atoms. As a result, not all molecules shown in Figure 2 are necessarily chemically viable; unfavorable bonding interactions, thermodynamic or kinetic instability, or the absence of feasible synthetic routes may preclude their existence. At the bottom of each couple are the corresponding cyclic isomers, among them ethylene oxide (**V**), cyclopropane (**VI**), and aziridine (**VII**)—three stable and strained cyclic compounds of both economic and practical interest [16, 17].

**FIGURE 3:**
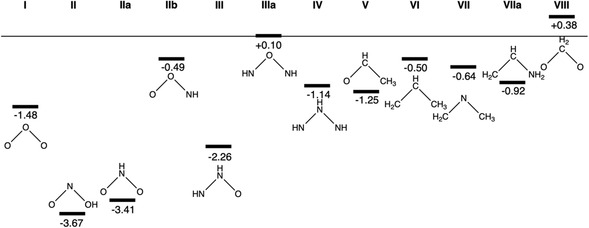
Energy gap between bent and cyclic three‐membered ring systems (in eV). All cyclic species are taken as zero (horizontal line) and the energy of their bent structures is shown in the graph. Computed at ZORA‐BLYP‐D3(BJ)/TZ2P level.

We have first computed the isomerization energies between the bent and cyclic molecular pairs, at the ZORA‐BLYP‐D3(BJ)/TZ2P level of theory with ADF [18, 19, 20, 21]. Although all constitutional bent isomers have been computed, only the most stable one is depicted in Figure 3. Isomerization energies support that the bent isomer is the most stable in almost all cases. For instance, if we go back to the three well‐known three‐membered rings ethylene oxide (**V**), cyclopropane (**VI**), and aziridine (**VII**), the bent isomers are more stable by 1.25, 0.5 and 0.4/0.9 eV, respectively (Figure 3).

Noticeably, in some cases, such as **II**, **III**, **VII**, and **VIII**, the same cyclic compound can drive to more than one different constitutional bent isomer, each possessing a double bond but differing in atomic connectivity. Based on general knowledge of Lewis structures for bent 18‐VE molecules (AX_2_E/AX_2_E_2_), the hierarchy flows like this: Primary factor is octet compliance—central atom must fit required bonds/lone pairs without hypervalency or radicals (e.g., N over C in H_2_CNCH_3_). Secondary is electronegativity (EN)—least EN wins ties by stabilizing δ^+^ charge centrally via Bent's rule hybrids. Tertiary is symmetry for minimal repulsions. For example, in the **II/IIa/IIb** set of pairs, **II** wins globally (no formal charges, −3.7 eV), despite **IIa** winning from EN plus symmetry (−3.4 eV); **IIb** fails worst (O^+^ central, most EN atom, −0.5 eV) . The **III/IIIa** pair nicely illustrates that, when octet rules are met, the least electronegative atom (N over O) best serves centrally—even with formal charge—as it stabilizes positive charge better while O terminals hold any negatives (−2.3/ + 0.1 eV, respectively). This also applies for **VII/VIIa**, when the isomer with the least electronegative central atom is favored, even at the expense of symmetry (−0.6/−0.9 eV, respectively).

One of the reviewers suggested including the Criegee intermediate H_2_CO_2_ (CH_2_OO) [22, 23, 24]. For this system, our computations and literature data agree that the cyclic dioxirane isomer is substantially more stable than any bent valence isomer, by 0.4 eV (**VIII**) and that among bent minima, bisoxy biradical structures lie below the zwitterionic O‐centered C=O^+^ form, by 0.7 (**VIIIa**) eV. This latter is the one referred as Criegee intermediate. ECSA supports the larger stability of the closed species [25, 26, 27]. Because the zwitterionic species involves a formal positive charge on oxygen and strong charge separation, it falls outside the closed‐shell 18‐valence‐electron manifolds considered in this work. Thus, CH_2_OO represents a distinct class of Criegee intermediates (ring and biradical dominated), rather than a genuine counterexample to the “bent double‐bond” stability pattern for closed‐shell 18‐VE isomers. The experimentally observed bent structure (∠OOC = 118°) reflects biradical character mixing that populates lone‐pair‐like orbitals on oxygen, stabilizing the hybrid. Overall, these examples illustrate that, although different constitutional isomers with a double bond can give rise to the same cyclic compound, with no double bond, their relative stabilities depend on a balance between molecular symmetry and the electronegativity of the central atom. In the case of Criegee intermediates (e.g., CH_2_OO), the cyclic dioxirane isomer unequivocally exhibits greater thermodynamic stability than the open bent zwitterionic form (H_2_C^+^–O–O^−^). This constitutes a unique exception among 18‐VE systems, where formal charge avoidance overrides bent geometry preferences. This singular case underscores formal charge minimization as the ultimate arbiter when octet compliance permits multiple viable Lewis structures.

On the other hand, as expected due to the presence of C=O, N=O, N=N or O=O double bonds, the bent molecular structures such as the acetaldehyde, the nitrite anion, triazene [28, 29], or ozone are capable of the UV absorption thanks to the *π* → π* and/or n → π* transitions in the range of 210–320 nm, ≈200 nm (N=O, nitrite), ≈170 (C=C, propene), ≈180 (C=N, acetone imine), ≈170 (C=C, propene), ≈140 (C=O, acetaldehyde), ≈190 (N=N, predicted for diethyldiazene), and ≈254 nm (O=O, Hartley band ozone) [30]. Opposite to these UV absorbing bent molecules, cyclopropane, ethylene oxide, and aziridine feature three‐membered ring structures and are UV silent because they lack π* orbitals and their electronic transitions are restricted to high‐energy *σ* → σ* excitations. This latter has been confirmed by TD‐DFT computations, showing the band for bent ozone at 253 nm, whereas there is not band for cyclic ozone (Figure S1). These characteristics turn out to be notable among three‐atom molecules with 18 VEs, where the molecular geometry significantly influences their electronic properties and interactions with UV radiation. Thus, the 18 VEs number defines both the capacity of UV absorption for bent molecules and the lack of UV absorption for triangular cyclic molecules [31]. This is equivalent to having the pair in light‐absorbing/non‐light‐absorbing states. Finally, the triborane B_3_H_9_ also meets the required criteria of having three second‐row atoms and 18 VEs. Importantly, it serves as compelling evidence that the tension attributed to triangular cyclic molecules is only partially valid, as the electronic cloud between atoms is not linear but rather curved, as demonstrated by the out‐of‐line position of the bridging hydrogen atoms. However, no bent molecule associated with this borane is suggested.

### 2.2 Is the Trend Kept from 18‐VEs Bent Triatomic Molecules to 24‐, 30‐, or 36‐VEs?

Among the discussed above systems enclosed in Figure 2, the bent systems are confirmed to be more stable than their cyclic isomers. Aside from **IIIa**, where bent and cyclic forms are nearly equal in energy, and cyclic **VIII** for which the cyclic form is the most stable, the cyclic‐bent gap ranges from 0.5 eV for **IIb** to 3.7 eV for **II**, supporting the preference for the isomer with the least electronegative central atom, as noted above (Figure 3). Moreover, substituting an atom in ozone (**I**) by a less electronegative N atom (**II**) increases the gap from 1.5 to 3.7 eV, whereas for **VI**, introducing an N atom (**VII** or **VIIa**) results in a much smaller increase, from 0.5 to 0.6 or 0.9 eV. Thus, the position of the atoms clearly affects this gap. These large gaps justify the relevance of the 18‐VEs molecules.

**FIGURE 4:**
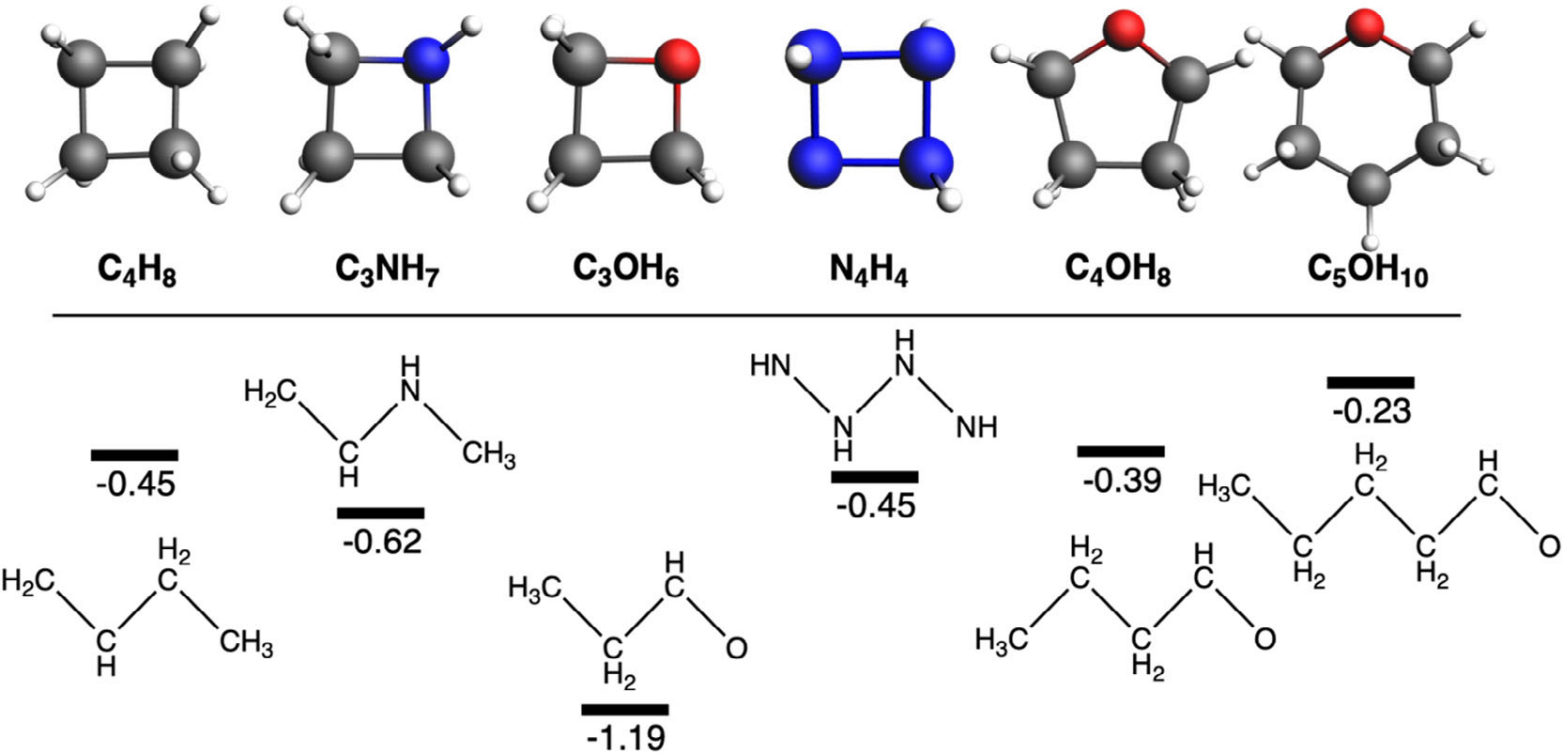
Energy gap between bent and cyclic four‐, five‐, and six‐membered ring systems (in eV). Influence of increasing electronegativity on ring strain in four‐membered cycles. Comparison of strain relief as ring size increases from square to pentagon to hexagon in C_*x*_O skeleton (*x* = 3–5) geometries. Computed at ZORA‐BLYP‐D3(BJ)/TZ2P level.

Next, if we move to 24‐VE systems, this gap is reduced, and such reduction continues in case of 30 and 36 valence electrons molecules (Figure 4). In particular, if we move from C_2_OH_4_ to C_3_OH_6_ to C_4_OH_8_ to C_5_OH_10_, the gap decreases from 1.3 to 1.2–0.4 to 0.2 eV, respectively. The reason must be found in the decrease of strain, i.e., the larger the ring size, the smaller the ring strain, and thus the smaller the cyclic‐bent gap. This is the reason of the uniqueness of such 18‐VEs compounds. This strain is further illustrated by comparing cyclic C_4_H_8_ and N_4_H_4_, both 24‐VEs systems in a square geometry: despite the increased electronegativity difference between carbon and nitrogen, the energy gap between the bent and cyclic forms remains unchanged (0.5 eV, Figure 4). This invariance demonstrates that electronic polarization, rather than electronegativity effects, is the primary source of ring strain.

### 2.3 Stratospheric Constraints: Ozone Emerges as the Sole Functional 18‐Electron Compound

Among the 18‐VEs systems discussed above, ozone stands out as one of the most extensively studied triatomic molecules. Most noticeably, despite its low concentration, ozone (O_3_) uniquely protects Earth from harmful UV radiation because it is the only feasible 18‐valence‐electron compound capable of UV filtration in the hydrogen‐poor stratosphere (further described in Discussion S1 in SI) [32]. In 1913, it was confirmed by Fabry and Buisson that ozone was the UV‐absorbing agent [33, 34], but it was in the period 1928–1958 that Dobson maps the stratospheric ozone distribution and quantifies its shielding effect [35]. Finally, in 1985, the Antarctic ozone hole was discovered, which highlighted ozone's irreplaceable protective role [36]. Sydney Chapman established the first mechanistic framework for stratospheric ozone formation (1930) known as the ozone‐oxygen or Chapman cycle by which ozone absorbs UV light that is released as heat [37, 38]. In particular, it is accepted that two main processes occur

(1) $$O_{2}+\mathrm{hv}\;\to \;2O$$

(2) $$O+O_{2}+M\;\to \;O_{3}+M$$

(3) $$O_{3}+\mathrm{hv}\;\to \;O_{2}+O$$

(4) $$O_{3}+O\;\to \;2O_{2}$$

First is the ozone formation (Equations (1) and (2)), and second is the ozone decomposition or ozone‐oxygen cycle (Equations (3) and (4)). The first process is made of two steps: the first step is the generation of atomic oxygen achieved by homolysis of O_2_ induced by UVC light with *λ* < 242 nm (Equation (1) ), thus preventing UVC light reaching the Earth, and the second step is the reaction of atomic oxygen with O_2_ to produce ozone (Equation (2)). The second process is the ozone‐oxygen cycle, which begins with the photodissociation of ozone (Equation (3)). When ozone absorbs a photon in the 240–310 nm range (UVB), it becomes excited and dissociates into O_2_ and atomic oxygen (O). This process absorbs ~95% of incoming UVB radiation. Subsequently, the atomic oxygen reacts with ozone (O_3_) to regenerate ozone (O_2_) (Equation (4)). Meanwhile, UVA radiation (315–400 nm) is not absorbed by ozone, allowing 90–95% of it to reach Earth's surface.

**FIGURE 5:**
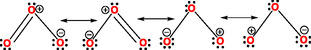
Resonance structures of bent ozone.

Unlike many existing studies that prioritize kinetic analyses [39], we focus on bonding structures, offering a complementary perspective while retaining respect for physical approaches. Crucially, we identify the O=O double bond (as illustrated in the Lewis structures of Figure 5) as central to ozone's UV absorption [40]. Ozone exhibits resonance between its four canonical forms, the two major contributors are the two on the left. Their existence makes that the O=O double bond absorption in O_3_ is red‐shifted compared to O_2_ due to broader π‐electron delocalization, which facilitates *π* → π* transitions in the UVB spectrum. This explains why UVC (100–280 nm) is primarily absorbed by O_2_, driving its photodissociation, while UVB (280–315 nm) is absorbed by ozone (O_3_), leading to ozone photodissociation or from our view excited ozone, O_3_*. Ozone displays two bands in the UV that are commonly known as the Hartley (200–310 nm, peaking at ~ 260 nm) and Huggins bands (310–370 nm, peaking at ~327 nm) that are key in the ozone‐oxygen cycle. In their absence, the UVB rays would reach the Earth surface penetrating the troposphere. The Chapman cycle's reliance on ozone dissociation (O_3_ + hν → O_2_ + O) and atomic oxygen's rapid recombination (O + O_2_ + M → O_3_ + M) cannot fully account for the observed stratospheric O_3_/O ratio, ≈5 × 10^5^ at 25 km or 100 at 40 km as this mechanism inherently requires a steady O‐atom supply that would, in theory, deplete ozone faster than observed [37, 41]. This paradox suggests either (1) unaccounted O‐atom sinks (e.g., catalytic removal via NO_*x*_/Cl), or (2) alternative ozone regeneration pathways bypassing atomic oxygen entirely—a gap where cyclic ozone (triangular O_3_) could play a critical role. If metastable c‐O_3_ forms through UV‐excited state rearrangements (O_3_* → cyclic‐O_3_), its distinct photochemistry might enable direct O_3_ reformation (e.g., c‐O_3_ → b‐O_3_ + heat) or energy‐transfer reactions (c‐O_3_ + N_2_ → b‐O_3_ + N_2_*) without O‐atom intermediaries. Such pathways could decouple ozone production from atomic oxygen availability, helping sustain high O_3_ concentrations [39, 42, 43, 44, 45].

### 2.4 Complementary Mechanism for the Decomposition of Ozone to Chapman's One

On this basis, we can describe a parallel cycle to Chapman's cycle that does not require atomic oxygen [37]. For this to be possible, it is required that the bent isomer (b‐O_3_) is more stable than the cyclic isomer c‐O_3_. All this occurs for any of the bent/cyclic couples studied in this work as shown in Figure 3. If we now restrict our discussion to the bent ozone (b‐O_3_) and cyclic ozone (c‐O_3_) pair, the energy absorbed by the bent form falls between 240–300 nm (5.17–4.13 eV), which is significantly higher than the energy difference between the ground state b‐O_3_ and c‐O_3_ (1.48 eV, or 34.1 kcal·mol^−1^). The triplet state of b‐O_3_ is 0.98 eV (22.5 kcal·mol^−1^) above the ground state, as computed at the ZORA‐BLYP‐D3(BJ)/TZ2P level [18, 19, 20, 21]. Hoffmann and coworkers report a value of 22.7 kcal·mol^−1^ (0.98 eV) for O_3_ ring opening and 46.6 kcal·mol^−1^ (2.02 eV) for dissociation of the cyclic form into O_2_ and O [46, 47]. These isomerization energies are consistent with those obtained from higher‐level multireference methods. Notably, all these energy values are quite small compared to the energy absorbed by ozone, even at 360 nm (3.44 eV), which lies well within the UVA region.

**FIGURE 6:**
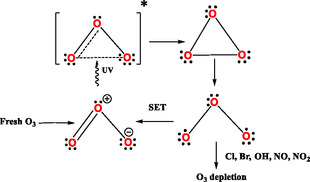
Proposed bent‐cyclic ozone cycle with no atomic oxygen participation.

**FIGURE 7:**
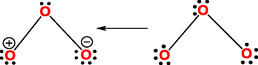
The biradical O_3_ form by single electron transfer SET converts to one of the resonant forms of b‐O_3_.

Thus, in this work, we propose a parallel cycle to the traditional explanation of the direct decomposition of ozone into atomic and molecular oxygen (Figure 6) [46, 47]. Instead of an immediate breakage of the ozone molecule by the action of UV light into O_2_ and O, we suggest that the absorption of a photon excites b‐O_3_ to an excited state. This excited form of O_3_ could, upon relaxing, transition to c‐O_3_ that itself could transition to a bent triplet state in which the terminal oxygen atoms possess each an unpaired electron. The depletion of b‐O_3_ caused by catalytic destruction processes involving radicals like hydroxyl (OH), nitric oxide (NO), chlorine (Cl), and bromine (Br) can easily be explained by reaction of the long‐lived triplet state with these radicals. However, for most of the occasions, the molecule in the triplet state would gradually relax into the common angular form of O_3_, b‐O_3_, with a double bond between the terminal oxygen atoms ready to absorb a UV‐photon. We propose this cycle to be named as the bent‐cyclic ozone cycle (Figure 6). It consists of 5 steps:

‐ Step 1: Excitation—Bent ozone absorbs UVB, leading to an excited‐state transition that on relaxation leads to a cyclic c‐O_3_.
‐ Step 2: Homolytic Cleavage—The triangular ozone undergoes homolytic cleavage of one sigma bond, forming a bent triplet biradical (·O‐O‐O·).
‐ Step 3: Relaxation—The biradical relaxes back into b‐O_3_ by a SET process, completing the cycle. Meanwhile, newly formed b‐O_3_ produced by the Chapman reactions (reactions 1 and 2) enters the cycle, replenishing the ozone that is lost when radicals in the stratosphere react with the diradical form of ozone (Figure 7).
‐ Step 4: Radical Interactions—The intermediate biradical ·O‐O‐O· eventually may interact with stratospheric radicals (e.g., NO·, OH·, Cl·), influencing ozone depletion pathways.

**FIGURE 8:**
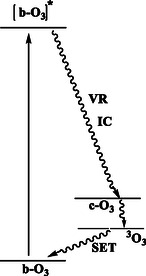
New proposed photochemical process: bent‐cyclic ozone cycle.

Atomic oxygen plays no direct role in this process, except indirectly through a side mechanism where ozone‐depleting radical reactions facilitate the introduction of fresh O_3_. A detailed representation of this photochemical cycle is shown in Figure 8. Here, b‐O_3_ absorbs UVB radiation via its delocalized O=O double bond, forming an excited state ([b‐O_3_]*). Through vibrational relaxation (VR) and internal conversion (IC), [b‐O_3_]* transitions to c‐O_3_, followed by homolytic cleavage of one O—O bond. This generates two terminal oxygen atoms, each retaining an unpaired electron, which adopt either a singlet (b‐^1^O_3_) or triplet (b‐^3^O_3_) spin state. While these diradicals can react with stratospheric radicals (e.g., NO_*x*_, Cl_*x*_) to deplete ozone, most revert to b‐O_3_ via Single Electron Transfer (SET), reinitiating the cycle. Notably, atomic oxygen does not participate in this mechanism.

The above newly proposed mechanism complements the Chapman cycle rather than contradicting it [37]. The Chapman cycle is essential for the efficient production of ozone (O_3_) in the stratosphere, as it consumes harmful UVC radiation by homolytically splitting molecular oxygen (O_2_) to produce highly energetic atomic oxygen (O). This atomic oxygen then reacts with additional O_2_ to generate ozone. In this process, one atomic oxygen atom results in the formation of one ozone molecule. According to Chapman, however, under UVB radiation, each ozone molecule decomposes to yield one atomic oxygen atom and one oxygen molecule. Despite this, experimental observations show that the ratio of ozone to atomic oxygen in the stratosphere is approximately 10^5^ to 1. This high ratio is difficult to explain using only the simple equations of ozone formation and decomposition described by the Chapman cycle.

The bent‐cyclic ozone cycle provides a simpler explanation for this observation. In this alternative pathway, atomic oxygen is not directly required for the regeneration of ozone, allowing the cycle to sustain a much higher ozone‐to‐atomic oxygen ratio. Nevertheless, some ozone is inevitably lost through reactions between the ozone biradical and other radicals present in the stratosphere. To offset this loss, fresh ozone must be continually supplied, which occurs via the Chapman mechanism‐specifically, through the reaction of atomic oxygen with molecular oxygen. Importantly, while the formation of new ozone relies on atomic oxygen, this process takes place outside the bent‐cyclic ozone cycle. As a result, the cycle itself can maintain a high ozone‐to‐atomic oxygen ratio, since its operation does not depend on the continuous presence of atomic oxygen. This distinction has important implications for our understanding of photochemical reactions, climate modeling, and the impact of atmospheric pollutants on ozone depletion.

*Noticeably*, to support this model, again TD‐DFT quantum chemical calculations have been performed to evaluate the excited‐state transitions of ozone under UV. In particular, the lowest excitation of b‐O_3_ corresponds to the singlet triplet excitation that amounts to 0.94 eV, i.e., a feasible value, and in agreement with the above discussion.

## 3 Conclusion

The Electronically Confined Space Analogy (ECSA) postulate offers a unified perspective for interpreting the structural and electronic properties of 18‐valence‐electron (18‐VE) systems composed of second‐row elements. By linking molecular geometry to electronic confinement, this study shows that bent isomers of triatomic 18‐VE molecules—such as ozone (O_3_), nitrite anion (NO_2_^−^), and acetaldehyde (H_4_C_2_O)—display strong UV absorption due to delocalized π systems, whereas their cyclic analogs (e.g., cyclopropane, ethylene oxide) are photochemically inert. This contrast is rooted in the presence of low‐energy *π* → π* or n → π* transitions in bent structures, which are absent in strained cyclic forms.

Our findings indicate that bent isomers are consistently more stable than their cyclic counterparts, with energy gaps ranging from 0.49 to 3.67 eV. This enhanced stability is primarily governed by electronic polarization and symmetry, rather than differences in electronegativity. The thermodynamic preference for bent structures ensures that UV‐absorbing species predominate under atmospheric conditions, playing a critical role in shielding biological systems from harmful radiation.

A central outcome of this work is a new rationale for ozone's unique role as Earth's stratospheric UV filter. The ECSA framework elucidates ozone's exceptional photochemical properties and its dominance among 18‐VE molecules, providing a molecular‐level explanation that complements—or even extends beyond—the traditional Chapman mechanism. While Chapman's model describes the photochemical formation and destruction of ozone, ECSA clarifies why ozone, among all possible 18‐VE species, is structurally and electronically optimized for effective UV absorption.

Building on this, we propose a UV‐driven ozone cycle that complements the Chapman cycle by focusing on ozone's interaction with UVB radiation. This model, supported by TD‐DFT calculations, highlights the feasibility of exciting bent ozone to a triplet state and accounts for the observed stratospheric [O_3_]/[O] ratio of approximately 10^4^:1. Unlike traditional models, which often assume immediate recombination of atomic oxygen with cogenerated O_2_, our approach incorporates intermediate excited states and cyclic species, offering a more nuanced view of ozone's photophysical dynamics.

This alternative framework not only addresses gaps in conventional descriptions—such as those based on Van Etten's direct decomposition pathway—but also opens new avenues for exploring how excited‐state transitions and intermediates influence ozone's atmospheric lifetime and UV‐protective function. These insights may ultimately enhance our understanding of ozone's resilience and its response to anthropogenic perturbations, with implications for both Earth and other planetary atmospheres where analogous 18‐VE molecules might play a similar protective role.

## 4 Computational Details

All calculations were performed with the ADF software inside the Amsterdam Modeling Suite [18, 19, 20, 21, 48] at the ZORA‐BLYP‐D3(BJ)/TZ2P level of theory. The geometry optimizations were carried out without symmetry constraints, and analytical Hessians were computed to characterize the optimized structures as minima (zero imaginary frequencies). UV‐Vis absorption spectra has been computed through TD‐DFT at the CAMY‐B3LYP/TZ2P level of theory [49] in vacuo.

## Supporting Information

Additional supporting information can be found online in the Supporting Information section. Cartesian coordinates and energies of all systems quantum chemically analyzed. **Supporting Fig. S1:** UV‐Vis spectra of cyclic ozone (in red) and bent ozone (in blue). Involved molecular orbitals of the main band of bent ozone are also included, whereas that for cyclic ozone is silent. Computed at TD‐DFT CAMY‐B3LYP/TZ2P level of theory in vacuo. **Supporting** **Table S2:** Cartesian coordinates (in Å) and electronic ADF energies (in kcal/mol) of all systems under analysis.

## Funding

This study was supported by MCIN/AEI/10.13039/501100011033 (Grant PID2022‐138861NB‐I00 and CEX2021‐001202‐M), Direcció General de Recerca, Generalitat de Catalunya (Grant 2021SGR442), and Severo Ochoa (Grant CEX2023‐001263‐S and CEX2023‐001263‐S).

## Conflicts of Interest

The authors declare no conflicts of interest.

## Supporting information

Supplementary Material

## Data Availability

The data that support the findings of this study are available from the corresponding author upon reasonable request.
