# Supplementary material for: The Electronically Confined Space Analogy Elucidates How Second‐Row Triatomic 18‐Valence‐Electron Molecules Shape Life and Light
Source: ChemistryOpen. 2026 Mar 10;15(3):e202500557. doi: 10.1002/open.202500557 (PMC12972594; doi:10.1002/open.202500557)
Supplement: Supplementary file 1 — Supplementary Material [file OPEN-15-e202500557-s001.pdf]

SUPPORTING INFORMATION

The Electronically Confined Space Analogy Elucidates How Second-Row Triatomic 18-Valence-Electron Molecules Shape Life and Light

Jordi Poater,*[a,b] Clara Viñas,*[c] and Francesc Teixidor*[c]

[a] *Departament de Química Inorgànica i Orgànica & IQTCUB, Universitat de Barcelona, 08028 Barcelona Spain. E-mail:* jordi.poater@ub.edu

[b] *ICREA, 08010 Barcelona, Spain*

[c] *Institut de Ciència de Materials de Barcelona, Consejo Superior de Investigaciones Científicas, 08193 Bellaterra, Spain. E-mail:* clara@icmab.es; francesc@icmab.es

Discussion S1. How does ozone stand alone in protecting Earth from UV irradiation?

Chemical composition of living organisms, atmosphere and troposphere. The distinct optical properties of the two 18-VEs ECSA isomers—where most of the UV-absorbing bent form is thermodynamically the most stable (lowest-energy configuration, Figure 3 in the manuscript), and the non-absorbing cyclic form is the highest-energy configuration—likely play a critical role in protecting living organisms. If the UV-transparent cyclic isomers were the ground state, this natural UV-shielding mechanism would fail, leaving biological systems vulnerable to harmful radiation. This is accomplished by forcing the molecule's naturally bent (angular) structure into a strained cyclic (triangular) form. This strain raises the energy of the molecule, making the cyclic form thermodynamically unstable and driving it to revert to the bent configuration—thereby dissipating the absorbed harmful radiation as heat. These 18-VEs partners could correspond to the two endpoints of an isomeric/tautomeric continuum, as evidenced in N_3H_3. Despite being simplified snapshots, they effectively illustrate this system's behavior.

Chemical composition of living organisms, atmosphere and troposphere. The acronym CHON refers to carbon (~18%), hydrogen (~10%), oxygen (~65%) and nitrogen (~3%), that altogether account for about 96% of the mass of most living organisms. To note is that all these elements but hydrogen are second row elements. Besides these, there are two third row elements: calcium (~1.5%) and phosphorus (~1%). In the case of the human body, almost 99% of its mass is made up of just six elements: O, C, H, N, Ca, and P.[1]

Having highlighted the importance of second-row elements in living organisms, we now turn our attention to the most abundant elements in the troposphere. The Earth's troposphere (dry air by volume) consists primarily of nitrogen (~78%), oxygen (~21%), and argon (~0.9%), together accounting for more than 99% of dry air. Trace gases of particular interest include carbon dioxide (~0.04%, or about 360–419 ppm), methane (1.7–1.9 ppm), nitrous oxide (0.3–0.33 ppm), hydrogen (~0.5 ppm), ozone (10–200 ppb, varying with altitude and conditions), and water vapor,[2] which ranges from 0.01% up to 4% or higher depending on humidity.[3] As in the overall atmosphere, two second-row elements, i.e., nitrogen (N_2) and oxygen (O_2) remain the dominant components in the troposphere. Its composition closely mirrors that of the stratosphere, albeit with a few notable differences. On the other hand, the Earth's stratosphere (dry air by volume) consists primarily of nitrogen (~78%), oxygen (~21%), and argon (~0.93%), collectively accounting for ~99.9% of dry air. Trace gases include ozone (O_3, 8–15 ppm at 15–35 km altitude), carbon dioxide (~0.04%, 412 ppm), methane (~1.8 ppm), hydrogen (0.5 ppm), nitrous oxide (N_2O, ~0.3 ppm), and water vapor (3–5 ppm). The only striking contrast between the two layers is the much greater abundance of ozone and the markedly lower presence of water vapor in the stratosphere relative to the troposphere.[4]

Ozone's role in protecting Earth from UV irradiation. Ozone's beneficial effects to protect Earth from harmful UV irradiation are especially impressive given how little ozone actually sits between the sun and Earth's surface.[5] Ozone's dominance in the stratosphere confirms its status as the only second-row triatomic molecule with 18 valence electrons capable of UV filtration.[6] From our perspective, the scarcity of water-derived hydrogen inhibits the formation of NH-bonded nitrogen compounds, even given nitrogen's abundance in the stratosphere. In the absence of these NH-bonded compounds, ozone remains the only viable agent for UV filtration. When analyzing 18-VE compounds made of elements abundant in the stratosphere, only oxygen, nitrogen, or their combinations with one hydrogen atom per nitrogen atom are plausible (Figure 2). Examples include O_3, O_2NH, $O(NH)_2$, or $(NH)_3$. Yet, given the minimal hydrogen availability in the stratosphere, ozone is the sole functional compound here. While this holds true for Earth's atmosphere, other planetary atmospheres might host ozone alternatives.

References

[1] M. A. Zoroddu, J. Aaseth, G. Crisponi, S. Medici, M. Peana and V. M. Nurchi, *J. Inorg. Biochem.* **2019**, *195*, 120-129.
[2] D. R. Bates and M. Nicolet, *J. Geophys. Res.* **1950**, *55*, 301-327.

[3] U. o. A. Department of Atmospheric Sciences in *Composition of the Atmosphere*, *Vol.* **2025**.
[4] N. O. a. A. Administration in *Layers of the Atmosphere*, *Vol.* **2025**.
[5] U. E. P. Agency in *Information on Ozone and Ozone Depletion*, *Vol.* **2025**.
[6] A. Match, E. P. Gerber and S. Fueglistaler, *Atmos. Chem. Phys.* **2025**, *25*, 4349-4366.

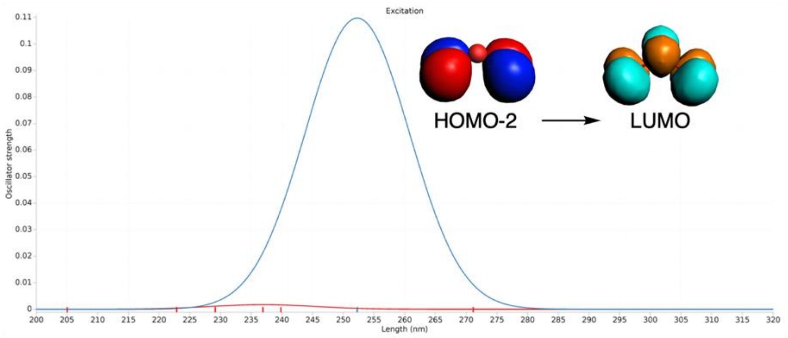

Figure S1. UV-Vis spectra of cyclic ozone (in red) and bent ozone (in blue). Involved molecular orbitals of the main band of bent ozone are also included, whereas that for cyclic ozone is silent. Computed at TD-DFT CAMY-B3LYP/TZ2P level of theory in vacuo.

Table S2. Cartesian coordinates (in Å) and electronic ADF energies (in kcal/mol) of all systems under analysis.

OOO closed (-259.1)

1	O	0.35110690	0.49076398	0.00000000
2	O	0.35110690	1.96146582	0.00000000
3	O	1.62475414	1.22611490	0.00000000

OOO bent (-293.3)

1	O	0.41443620	0.65112205	0.00000000
2	O	0.38987270	1.94497441	0.00000000
3	O	1.52266037	2.57062130	0.00000000

ONO closed (-420.1)

1	O	0.61110536	-2.75251428	0.76249329
2	N	-0.62083747	-2.11017956	1.22038436
3	O	-0.68156975	-3.49640826	0.76009684
4	H	-0.91922757	-1.58753377	0.36976685

ONOH bent (-504.7)

1	O	-2.50425078	-0.69823909	0.28407633
2	N	-1.65276202	-1.07647836	-0.42655537
3	O	-0.41761379	-1.45310115	0.37328996
4	H	0.18721614	-1.74285029	-0.34081092

ONHO bent (-498.6)

1	O	-2.58608903	-0.69882124	0.23924796
2	N	-1.52986375	-1.03320335	-0.30614531
3	O	-0.49367258	-1.45174241	0.21897947
4	H	-1.50985719	-0.95097178	-1.35450250

OONH bent (-431.5)

1	O	-2.64726761	-0.62218142	0.32360018
2	O	-1.64647694	-1.10195740	-0.38148709
3	N	-0.56244636	-1.41788810	0.27257948
4	H	0.03031488	-1.75407511	-0.51359987

NNO closed (-570.2)

1	O	0.58611221	-2.82102963	0.79778175
2	N	-0.67471628	-2.16610046	1.22067602
3	N	-0.71269731	-3.51635656	0.63476321
4	H	-0.89804379	-1.56834347	0.40473589
5	H	-0.74853651	-4.11830898	1.47645590

NHNHO bent (-622.3)

1	O	0.40290620	-2.86550512	0.56911673
2	N	-1.85762190	-2.80668395	1.01223194
3	N	-0.68270768	-3.30608064	1.02763844
4	H	-1.77282890	-1.89982258	0.51880170
5	H	-0.64375800	-4.22936260	1.50307723

NHONH bent (-567.8)

1	O	0.52588042	-2.74759483	0.12126771
2	N	-0.05217962	-2.15478424	1.17457811
3	N	0.83535257	-4.01769109	0.15292804
4	H	-0.14909410	-1.18921370	0.81457081
5	H	0.53394599	-4.34155966	1.10114477

NNN closed (-707.3)

1	N	1.03474378	0.50037316	-0.06855038
2	N	1.04175721	1.99014740	-0.02677904

3	N	2.33008356	1.24012840	-0.06146774
4	H	0.81376664	0.24804311	0.90761434
5	H	0.90124135	2.24572657	-1.01466522
6	H	2.65099167	1.29817688	0.91783703

NHNHNH bent (-733.6)

1	N	1.15625209	0.54147180	0.05323828
2	N	1.45896490	1.74845424	0.41672894
3	N	2.53333664	2.37019552	0.04256160
4	H	0.25851875	0.32504895	0.50391371
5	H	0.80155936	2.24744675	1.04594962
6	H	2.49638083	3.29617317	0.48685880

OCC closed (-840.9)

1	C	0.37530193	-0.04633968	0.13264532
2	O	0.36557025	1.39720926	-0.00996102
3	C	1.63954299	0.70333819	0.00781529
4	H	0.06374684	-0.41577579	1.11043775
5	H	-0.00926876	-0.59734702	-0.72664202
6	H	2.17498189	0.69783537	-0.94274227
7	H	2.24913254	0.88074293	0.89489556

OCHCH3 bent (-869.7)

1	C	0.55153255	0.20209602	0.23639318
2	O	0.27260623	1.37803939	0.10062511
3	C	1.90929323	-0.40137937	-0.03632288
4	H	-0.22321635	-0.52517253	0.58723746
5	H	2.27962827	-0.88984703	0.87633643
6	H	1.80995042	-1.19110099	-0.79527480
7	H	2.61791024	0.36001730	-0.37388951

CCC closed (-1069.2)

1	C	-0.30016625	-0.90165020	0.12624055
2	C	-0.30154659	0.61098578	0.01494787
3	C	1.01052795	-0.14648988	0.01494914
4	H	-0.55446491	-1.34206222	1.08641130
5	H	-0.55984227	1.18721923	0.89926710
6	H	-0.63510342	1.05255043	-0.92030046
7	H	1.63879500	-0.08206359	0.89922506
8	H	1.55975183	-0.21465092	-0.92027989
9	H	-0.63201674	-1.47640038	-0.73401206

CH2CHCH3 bent (-1080.7)

1	C	-1.05763944	0.00172177	0.45507495
2	C	0.21408245	0.40732480	0.41207648
3	C	1.30682385	-0.24545736	-0.38872311
4	H	-1.80412229	0.51744516	1.05466997
5	H	0.50914115	1.28180752	0.99685024
6	H	-1.39732793	-0.86643741	-0.10953277
7	H	2.13012191	-0.57013616	0.26270061
8	H	1.73893621	0.45967621	-1.11313335
9	H	0.93563983	-1.11819690	-0.93870153

CNC closed (-958.7)

1	C	-0.29045156	-0.88808099	0.12237854
2	N	-0.28340738	0.59954403	0.07387809
3	C	1.00022469	-0.15001840	0.00903983
4	H	-0.59181106	-1.30584540	1.08063685
5	H	-0.64057839	-1.43861803	-0.74954015
6	H	-0.54491580	0.91310941	-0.86435209

7	H	1.63508540	-0.03376462	0.88486998
8	H	1.53205992	-0.19613329	-0.94021274

CH2NCH3 bent (-973.5)

1	C	-0.37681566	-0.82227303	0.03866242
2	N	-0.07198171	0.37180905	-0.26770344
3	C	1.26849010	0.84682025	0.06767521
4	H	0.31768529	-1.51829183	0.54374624
5	H	-1.37419130	-1.19994612	-0.20251543
6	H	1.89416213	0.08900446	0.57154263
7	H	1.17883139	1.72967266	0.71277800
8	H	1.76743506	1.17419422	-0.85320097

CH2CHNH2 bent (-979.9)

1	C	-0.43528534	-0.92513683	0.06400711
2	C	0.06679628	0.25260101	-0.34217706
3	N	1.30182543	0.80148587	0.01875004
4	H	0.12994591	-1.60771985	0.69623620
5	H	-1.44107934	-1.21640042	-0.21763653
6	H	1.97938319	0.14911641	0.40383919
7	H	1.71830191	1.43169225	-0.65830202
8	H	-0.53035372	0.91362404	-0.97045939
